# Examining the news media reaction to a national sugary beverage tax in South Africa: a quantitative content analysis

**DOI:** 10.1186/s12889-021-10460-1

**Published:** 2021-03-06

**Authors:** Michael Essman, Fernanda Mediano Stoltze, Francesca Dillman Carpentier, Elizabeth C. Swart, Lindsey Smith Taillie

**Affiliations:** 1 Gillings School of Global Public Health, Department of Nutrition, University of North Carolina at Chapel Hill, Chapel Hill, NC USA; 2 Carolina Population Center, University of North Carolina at Chapel Hill, Chapel Hill, NC USA; 3 Hussman School of Journalism and Media, University of North Carolina at Chapel Hill, Chapel Hill, NC USA; 4 Faculty of Community and Health Sciences, University of the Western Cape, Robert Sobukwe Rd, Bellville, Cape Town, South Africa

**Keywords:** Sugar-sweetened beverages, Tax, Policy, South Africa, Obesity, Diabetes, News media

## Abstract

**Background:**

South Africa was the first sub-Saharan African country to implement a sugar-sweetened beverage (SSB) tax called the Health Promotion Levy (HPL) in April 2018. Given news media can increase public awareness and sway opinions, this study analyzed how the media represented the HPL, including expressions of support or challenge, topics associated with the levy, and stakeholder views of the HPL.

**Methods:**

We performed a quantitative content analysis of online South African news articles related to the HPL published between January 1, 2017 and June 30, 2019. We coded the presence or absence of mentions related to health and economic effects of the HPL and HPL support or opposition. Prevalence of these mentions, overall and by source (industry, government, academics, other), were analyzed with Pearson χ_2_ and post-hoc Fisher exact tests.

**Results:**

Across all articles, 81% mentioned health, and 65% mentioned economics topics. 54% of articles expressed support, 26% opposition, and 20% a balanced view of the HPL. All sources except industry expressed majority support for the HPL. Health reasons were the most common justifications for support, and economic harms were the most common justifications for opposition. Statements that sugar intake is not related to obesity, the HPL will not reduce SSB intake, and the HPL will cause industry or economic harm were all disproportionately high in industry sources (92, 80, and 81% vs 25% prevalence in total sample) (*p* < 0.001). Statements that sugar intake is related to obesity and non-communicable diseases were disproportionately high in both government (46 and 54% vs 31% prevalence in total sample) (p < 0.001) and academics (33 and 38% vs 25% prevalence in total sample) (*p* < 0.05). Statements that the HPL will improve health and the HPL will reduce health care costs were disproportionately high in government (47% vs 31% prevalence in total sample) (*p* < 0.001) and academics (44% vs 25% prevalence in total sample) (*p* < 0.05), respectively.

**Conclusions:**

Industry expressed no support for the HPL, whereas academics, government, and other sources mainly expressed support. Future studies would be improved by linking news media exposure to SSB intake data to better understand the effects news media may have on individual behavior change.

**Supplementary Information:**

The online version contains supplementary material available at 10.1186/s12889-021-10460-1.

## Background

Sugar-sweetened beverages (SSBs) are one of the largest global drivers of added sugar consumption and are independently associated with increased risk of obesity, diabetes, and cardiovascular diseases [1–4]. In an effort to reduce SSB consumption at the population level, several countries and municipalities have introduced SSB taxes since 2014 [5, 6]. Although African countries do not purchase SSBs at rates as high as Western countries like the US, Mexico, and Chile, SSB sales are rapidly increasing across Africa [7]. In particular, South Africa has one of the highest SSB sales rates in Africa [7]. Such high rates of consumption are occurring in an environment where type II diabetes is the greatest cause of death in South African women and the second leading cause of death overall [8]. If left unchanged, the growing SSB consumption is likely to increase the burden of obesity and chronic disease in the future [8–10]. In response to this public health challenge, on April 1, 2018, South Africa became the first sub-Saharan African country to implement a tax on sugary beverages, called the Health Promotion Levy (HPL), to reduce South Africans’ consumption of added sugar.

The HPL not only affects potential SSB consumers by causing price increases, but news media coverage of the tax can inform consumers as to the tax’s existence and purpose. News media can assist a public health intervention, through its ability to reach a wide public audience and second, through agenda setting, whereby news organizations control topic salience for the public and urgency for policy action through the frequency of news coverage [11–14]. By controlling the public’s frequency of exposure to the topic, news media affect how important the topic is perceived to be by the public. In addition to setting the public’s agenda, the way news media frame public policies related to health is important to the eventual effectiveness of such policies in reducing illnesses [15]. Framing defines how topics are understood by emphasizing certain aspects of the topic over others [16]. News media framing of obesity as a disease caused by environmental factors rather than poor individual choices may improve public acceptance of government intervention and accelerate the implementation of SSB taxes [17–19]. By defining a social problem and the dimensions along which it should be understood, framing of the SSB tax policy is crucial to whether the policy is ultimately passed and implemented [20]. News media can also influence public knowledge about SSBs and perceptions of risk, as well as their awareness and acceptance of the tax, which could affect dietary intake. For example, a recent study from Mexico found that increased SSB tax awareness was associated with increased odds of reducing SSB consumption after the tax [21]. Another study in Oregon, USA found that participants who were aware of the mass media campaigns were more likely to want to reduce their consumption and to agree that excessive sugar consumption leads to health problems [22].

Studying media frames related to public health polices is essential for understanding the arguments and strategies that stakeholders use to influence public perceptions and government policies [11]. Food and beverage industries have employed a playbook to resist regulation including neutralizing negative media coverage of their products and influencing the policymaking process due to economic power [23, 24]. In order to engage these policy criticisms and political influences, public health advocates can also use the news media to inform the public of their message [25]. While an extensive literature has examined the role of news media in other public health policies, such as alcohol and tobacco control [26, 27], evidence for news media responses to SSB policies remains scarce. Most of the current research comes from the United Kingdom (UK), where researchers found that the issue of sugar consumption as a problem for public health became increasingly discussed by the British media in the two years leading up to the passage of a UK SSB tax [17, 28, 29]. These articles have provided a systematic framework for understanding how news media in the UK changed leading up to the tax and after its passage, as well as identified the frames that the media has used to describe the problem of SSB consumption and the proposed solutions. In fact one article suggests major shifts in consumption in the UK as a result of this debate and law change in the two years before the SSB tax was initiated [30]. Developing an understanding of arguments that tend to support or oppose public health policies for obesity prevention will make it easier to identify communication strategies that are most successful. For example, Buckton and colleagues [17] found that overall coverage of the UK SSB tax was favorable, and the problem of SSB consumption was largely attributed to industry actions. However, passage of the SSB tax legislation was also met with a corresponding increase in negative media coverage [17]. Despite the developing evidence from the UK, it is unclear whether national news media outlets in countries like South Africa will vary in their responses to new SSB taxes. This is important to understand, because how SSB news is reported in the media may affect an individual’s response to the tax (perhaps via increased awareness of the health harms of SSBs), as well as governmental responses, such as whether politicians choose to discard, maintain, or strengthen the tax over time. Finally, according to the All Media Products Study 2015 by the South African Audience Research Foundation (SAARF), newspapers remain an important source of information in South Africa, as nearly half of adults (43.8%) read newspapers [31].

This is the first study of the news media response to an SSB tax in either a low- and middle- income country (LMIC) or African country. The purpose of this study is to examine the framing of online news articles related to the South African HPL before and after it was passed, how different stakeholder perspectives were portrayed in the news media discussion of the HPL, and the association between proposed solutions for high sugar intake and the stakeholders deemed most responsible for carrying out those solutions. By categorizing statements made about the HPL in the news media and linking those statements to attributed stakeholder groups, we can begin to develop an evidence base for how different actors are responding to the public health policy.

## Methods

We performed a quantitative content analysis of South African newspapers, following previous methods developed for systematic search and coding of news media content related to SSB taxes [17, 28]. Our article search strategy, codebook development, and analysis are outlined below.

### Sample selection

#### Search strategy

We selected online news articles covering the HPL using two databases: (1) Nexis Uni and (2) ProQuest Central, both global databases that provide access to full text news articles. We searched for articles using the following search string:

("Sugary beverages" OR "sugar-sweetened beverages" OR "sugar sweetened beverages" OR “health promotion levy”) AND (Levy OR Levies OR Tax OR Taxes OR Taxation OR Legislat*) AND (“South Africa” OR “Eastern Cape” OR Free State OR Gauteng OR “KwaZulu-Natal” OR Limpopo OR Mpumalanga OR “North West” OR “Northern Cape” OR “Western Cape” OR Bhisho OR Bloemfontein OR Johannesburg OR Pietermaritzburg OR Polokwane OR Nelspruit OR Mahikeng OR Kimberley OR Cape Town OR Port Elizabeth OR Durban OR Rustenburg OR Soweto OR Pretoria OR “Mitchells Plain” OR Umlazi OR Katiehong OR Tembisa OR Khayelitsha OR Soshabguve OR Mamelodi OR Ibhayi OR Tshivhase OR Sebonkeng OR Mabopane OR Chatswork))

South African city search terms were included to potentially pick up smaller, local papers published in South Africa. We did not restrict to particular newspapers and included any South African newspaper source captured by the Nexis Uni or ProQuest Central databases. Additional File 1: Table S1 displays the study sample with circulation numbers, which includes most of the major English language newspaper publishers in South Africa [32].

#### Eligibility criteria

Articles were included for analysis if they were published between January 1, 2016, one month prior to the initial announcement of a plan to tax SSBs by Finance Minister Pravin Gordhan, and June 30, 2019, when the article search was conducted. All articles were published in English, which is the primary language in South African education, journalism, broadcasting, and advertisements [33].

**Fig. 1:**
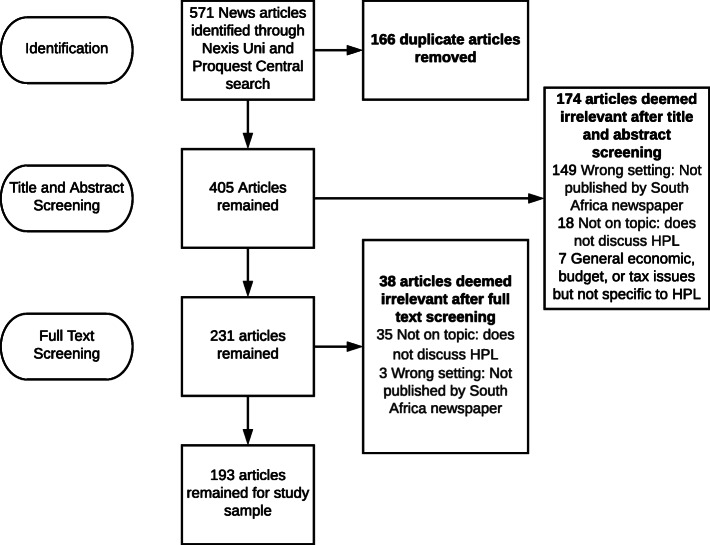
Flowchart for article selection process

To be included in the search, articles must have included discussion of the South African HPL. Discussion of the HPL was defined as including at least one of the following topics: the potential effects of the HPL (either on health, economics, or SSB consumption), statements of support or opposition toward the HPL, explanations of the purpose of the HPL (to reduce consumption of unhealthy products or to improve health), or other statements that explain a purpose, goal, or likely outcome of the tax. Articles were excluded if they were duplicates of previous articles found in the search, if they were not relevant to the HPL (e.g., if they were about Value Added Taxes in South Africa or about general tax policy without any specific discussion of the HPL and its purpose or consequences), or if they were not published by a South African news source. Articles that discussed SSB consumption but did not discuss the HPL were excluded. Given our focus on news media, we also excluded reports from non-governmental organizations (NGOs), law reviews and journals, and government documents. Articles could either be news articles written by journalists or opinion letters written to the newspaper and subsequently published, as publishing opinions was considered a view of the HPL presented by the newspaper. A flowchart for the article selection process is depicted in Fig. 1.

#### Data extraction

Articles from the initial search were downloaded to the online software product Covidence [34]. Two investigators (ME, FM) independently screened the article headline and first paragraph of the full text and excluded irrelevant articles using the inclusion and exclusion criteria. Disagreements were resolved after discussion of the inclusion/exclusion criteria between the two coders. Second, authors ME and FM independently screened the full text of relevant articles and eliminated those that did not fit the eligibility criteria. Articles were screened independently, with disagreements resolved after discussion between the two coders.

#### Final study sample

After the initial article search identified 571 articles, our final analytic sample included 193 articles published by the newspapers with the largest readerships in South Africa (Additional File 1: Table S1) [31].

### Coding

#### Codebook

To achieve inter-rater reliability, investigators (ME and FM) used articles written about the UK SSB tax to refine codebook definitions. This training set of articles from the UK context was used to decide which topics would be included in our study, and the codebook was updated in an iterative process until the two coders reached high agreement in understanding and using the codes. After the codebook training, the final set of definitions was established (Additional File 2: Table S2), and ME and FM coded a random subsample of 42 articles (22% of full sample) to ensure inter-rater reliability. We used Gwet’s AC1 as a measure for inter-rater agreement, as it has been shown to be a more stable measure of reliability than Cohen’s Kappa in instances of skewed distributions (in this case, many values of zero) [35, 36]. Items that achieved an acceptable Gwet’s coefficient were retained for the analysis, range = 0.77–0.97. After establishing acceptable inter-rater reliability and agreement, ME coded the remaining articles. Articles were coded by entering the data into a Microsoft Excel spreadsheet [37].

#### Outcomes: topics mentioned

Topics analyzed included two major categories—health and economics. Health topics included statements that sugar consumption is (or is not) related to obesity, sugar consumption is (or is not) related to diabetes, sugar consumption is (or is not) related to NCDs, and the HPL will (or will not) improve health outcomes. Economics topics included statements that the HPL will (or will not) cause industry or economic harm, the HPL will (or will not) reduce health care costs, and the HPL will (or will not) economically harm the poor. We added two additional policy-relevant topics: changes in SSB consumption and SSB reformulation as a consequence of the HPL, as these are both important goals of the HPL. All topics used in our analysis are listed and defined in Additional File 2: Table S2.

#### Outcomes: sources

We expanded upon the codebook used by Buckton and colleagues, which identified key topics and phrases in the articles analyzed [17]. Our study added to this approach by also including the source attributed to each topic mentioned. For example, if a statement was written that sugar consumption is not related to obesity, and this was attributed to someone working in the beverage industry, then the “industry” source was linked to this statement. Linking sources and topics allowed us to identify how different stakeholder perspectives were portrayed in the news media discussion of the HPL. We categorized sources as any person other than the journalist whom the journalist paraphrased or quoted as giving a statement about the HPL. Statements for which no source was given were attributed to the journalist. We categorized six source types as follows: industry, government, academics and medical experts, economists, NGOs, or private citizens. Industry representatives included leaders of SSB companies, leaders of trade organizations with workers in either the SSB industry or sugar growing industry, or any other representatives of companies in the SSB production or sales supply chain. Government representatives were defined as members of the South African parliament or any other government job relevant to the HPL, such as the Minister of Finance or Minster of Health. Academics and medical experts (henceforth referred to as academics) included persons with an academic or public health research job at a college or university. Medical experts in this category also included medical doctors, nurses, or other health professionals. Economists were sources referred to as such in the article, or any employee of a research organization or other company conducting economics research. NGO representatives were defined as sources from a non-governmental organization such as the World Health Organization or other relevant NGOs. Private citizens were defined as South African citizens or members of the public who expressed a view about the HPL that did not belong in any of the other aforementioned categories. Sources were categorized based on how they were described in the article or by searching for biographical information about the source or author if no description was given. All sources used in our analysis are listed and defined in Additional File 2: Table S2.

#### Outcomes: support or opposition

Statements expressing support or opposition to the HPL were categorized in the same manner as topics described above, with a support or opposition statement and the source. We also coded articles for whether (1) individual mentions of support or opposition were present in the article and (2) if the article as a whole supported or opposed the HPL (article-level support or opposition). We recorded individual mentions of support or opposition because news media reports can often include a variety of perspectives, and we wanted to capture all unique views expressed in each article and by each source. However, in our analysis, we were also interested in the number of *articles* published over time that were primarily in support of or opposition to the HPL. We classified articles as being in support of the HPL if they contained a greater number of supporting mentions than opposing mentions. Articles with more opposing mentions compared to supporting mentions were classified as opposing articles. Articles with an equal number of supporting and opposing mentions were classified as balanced articles. Supporting mentions were defined as statements noting that obesity is related to SSB consumption; the HPL will improve health outcomes; the HPL will reduce SSB consumption; the HPL will reduce health care costs; the HPL will not harm industry; the HPL will benefit the health of the poor. Opposing mentions were defined as statements that obesity is not related to SSB consumption; the HPL will not improve health outcomes; the HPL will not reduce SSB consumption; the HPL will not reduce health care costs; the HPL will cause industry or economic harm; the HPL will economically harm the poor.

#### Outcomes: proposed solutions

Proposed solutions were recorded if (1) there was a health problem described that was linked to sugar consumption (e.g., obesity, diabetes, other chronic diseases) and if (2) any of our key sources proposed a solution to this health problem (e.g., government should tax SSBs, industry should voluntarily reformulate their products, individuals should exercise more).

Proposed solutions were classified into four levels at which interventions could operate: changes in individual action, changes in individual beliefs, changes in the nutritional composition of food (e.g., reformulation of products or the introduction or removal of products), or other food environment-related changes. Individual actions included diet changes, exercise changes, or seeking education or counseling or other health information. Individual beliefs included interventions, initiatives, or structural responses proposed whose first steps are intended to change how individuals think about food, including public health campaigns, educational initiatives, marketing restrictions, and nutrition labels. Changes in nutritional composition of food included environmental or structural measures that would change the composition of foods available in the food supply, such as proposals to specifically incentivize reformulation or voluntary industry agreements for product reformulation. These proposed solutions for voluntary product reformulation differed from the topic code “reformulation as consequence” in that the voluntary solutions were proposed as a response to the health problem mentioned in the text, whereas reformulation as a consequence was mentioned as a direct consequence of the HPL. The fourth category included environmental or structural measures proposed that change the affordability, accessibility, or availability of foods, such as school food restrictions, taxes on unhealthy foods, subsidies of healthy foods, or restrictions on using financial assistance programs to purchase foods. The full definitions of our proposed solutions are provided in Additional File 2: Table S2.

We examined solutions by level of intervention, by source proposing the solution, and by the actor most responsible for carrying out the solution. For example, an academic source proposing SSB taxes as a means of reducing sugar consumption would be classified as an intervention related to changes in the food environment, suggested by academic source, and carried out by government regulation. Actors responsible for carrying out the proposed solutions included industry, government regulation, NGOs, and private citizens. This classification allowed us to separate an intervention into its component parts. Without this classification, government regulations would have all been categorized as the same.

### Analysis

#### Supporting, opposing, and balanced articles published before and after HPL passed

First, to understand trends in article stance on the HPL over time, we conducted a descriptive analysis of the total number of supportive, opposing, or balanced articles published during our search timeframe, as well as before and after HPL implementation. A Pearson χ_2_ test was used to evaluate whether the proportion of pro, con, and balanced articles differed by whether articles were published before versus after the tax was passed. To contextualize trends over time, we identified the timing of publication relative to additional key events, including the announcement of the South African Government’s plan to tax SSBs (February 2016), the publication of a key research article showing the two-year impact of an SSB tax in Mexico (February 2017), and the South African government passing the bill to tax SSBs (December 2017).

#### Topic mentions before and after HPL passed

Next, we analyzed the relative frequencies of topic mentions according to whether they were published before or after the HPL. Fisher’s exact tests were used to determine if the number of topic mentions differed based on being published before or after the HPL was passed, with a threshold for statistical significance set at 5% (α = 0.05).

#### Topic mentions by source

Next, we examined whether the proportion of topic mentions and support/opposition for HPL differed by source. We stratified by the proportion of those topic mentions that came from our six sources in addition to journalist opinion (statement attributed to the journalist). A Pearson χ^2^ goodness-of-fit test was used to determine whether the distribution of sources within topic categories was different from the distribution of sources within all topics (i.e., the expected distribution assuming no relationship between topic and source), setting our significance level at 5% (α = 0.05). We pooled all articles across time for this analysis because only 23% of our study sample was published after the HPL was implemented, which would leave many cell sizes small (less than 5), and therefore make statistical testing of differences between topics by source unreliable.

#### Supporting or opposing views of HPL by source

Because articles could contain more than one opinion of support or opposition from more than one source, we analyzed the total supportive or opposing views as the unit of analysis to characterize the perspectives presented from each of our six sources. Articles were analyzed according to total supporting and opposing opinions presented by each source. Fisher’s exact tests were used to determine if the number of supporting versus opposing views of the HPL within each source group were different, with a threshold for statistical significance set at 5% (α = 0.05).

#### Proposed solutions: actor responsible and level of action

To analyze the solutions proposed to solve health problems related to excess sugar intake, we first calculated the proportion of solutions that would be carried out by each actor as well as the sources that suggested that these actors should be responsible for carrying out the solutions. Next, we calculated the proportions of each level of action by the source suggesting the solution. Again, we used a χ^2^ goodness-of-fit test to determine whether these distributions differed significantly (α = 0.05) from the expected distributions based on the total number of sources mentioned in the study sample. Post-hoc Fisher’s exact probability z tests were used for pairwise comparisons between the percent contribution to solutions by source and the expected percent contribution based on overall prevalence of the source in the study sample.

All statistical analyses were performed in Stata 16 [38].

## Results

### Supporting, opposing, and balanced articles published before and after HPL passed

**Fig. 2:**
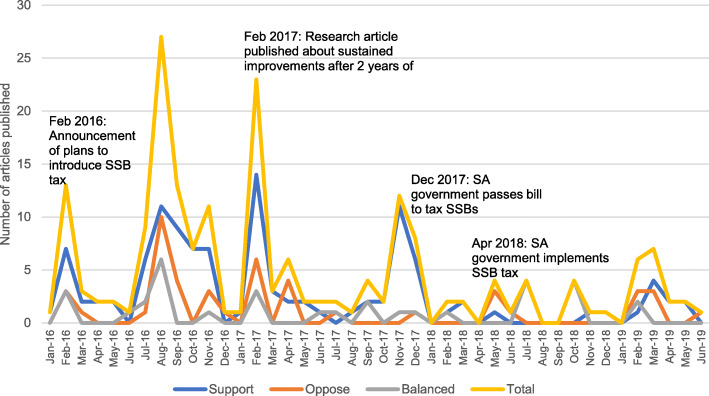
Prevalence of support, opposition, or balanced article coverage of HPL beginning with government announcement in 2016

Coverage of the HPL increased alongside three key events: after the Finance Minister’s announcement of a plan to tax SSBs, after the publication of a now widely cited research article on the two-year effects of a national SSB tax in Mexico [39], and the South African parliament’s passage of the bill to tax SSBs. Overall tax coverage decreased after passage of the bill in December 2017 (Fig. 2). Of all articles published in our sample frame, 54% were in support, 26% were in opposition, and 20% presented a balanced number of opinions (Fig. 2). There was no statistically significant difference in the proportion of articles expressing pro, con, or balanced stances towards HPL before vs. after the HPL was passed.

### Topic mentions before and after HPL passed

**Fig. 3:**
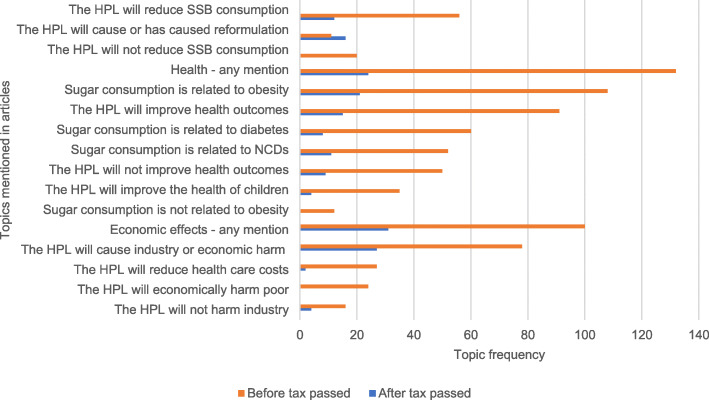
Frequency of total articles (*n* = 193) that mention any of the topics. Orange bars indicate articles published before HPL was passed, and blue bars indicate articles published after HPL was passed

Across the entire sample period, 82% contained any mention of health-related topics and 68% contained any mention of economics-related topics. The most common topics mentioned were: sugar consumption is related to obesity (79.8%), the HPL will improve health (69.9%), and the HPL will cause industry or economic harm (59.6%) (Fig. 3). A third of the articles suggested there would be no effect of the HPL on health. There were no statistically significant differences in the proportion of articles mentioning most topics before and after the HPL was passed. However, the proportion of articles mentioning that the HPL causes reformulation increased from 7% before to 36% after the HPL was passed (*p* < 0.001).

There were zero mentions that sugar was *not* related to diabetes or *not* related to NCDs, that the HPL would *not* reduce health care costs, and few statements were made directly denying economic harm to the poor—which made coding inter-rater agreement unreliable due to small sample size—so these topics are not presented in Fig. 3 even though they were searched for in our coding process.

### Topic mentions by source

**Fig. 4:**
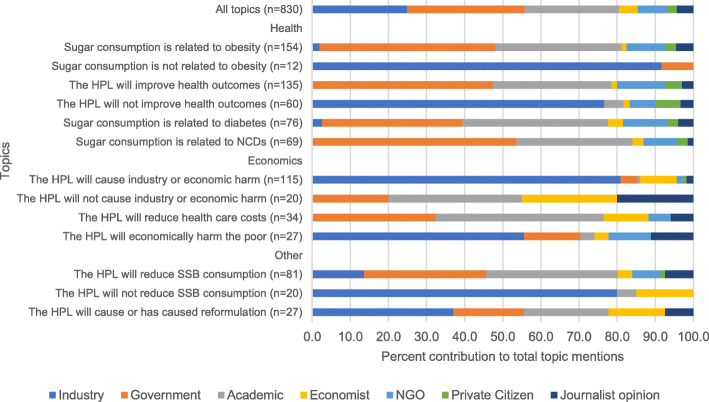
Percent contribution of each source to the total topic mentions, pooled across entire study timeline

The three most common sources cited in the news articles across all topics and across the sample period were industry (25%), government (31%), and academics (25%) (Fig. 4). Compared to all sources combined, the industry had a higher percentage of statements that sugar consumption is *not* related to obesity; HPL will *not* reduce SSB consumption; and HPL will cause industry or economic harm. Compared to all sources combined, government sources had a disproportionately higher percentage of statements that sugar consumption is related to obesity; HPL will improve health; and sugar consumption is related to NCDs, while academic sources had a disproportionately higher percentage of statements that sugar consumption is related to obesity and diabetes and that the HPL will reduce health care costs. When analyzed over the entire time period, there was no statistically significant relationship between source and mentions that HPL caused reformulation or that HPL will reduce SSB consumption, suggesting that each source mentioned these topics in proportion to their overall prevalence in the study sample. However, when analyzed by whether mentions were published before or after the HPL was passed, both industry and economists were significantly more likely to mention that reformulation would result from the HPL after the tax was passed compared to before.

### Supporting or opposing views of HPL by source

**Fig. 5:**
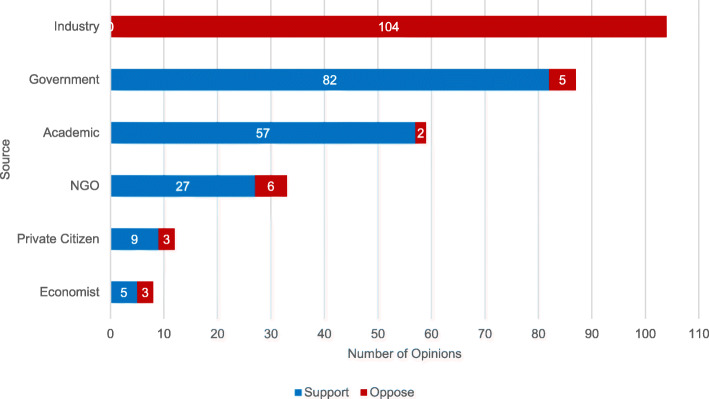
Mean frequency of supporting or opposing opinions of the HPL by Source

Among all articles, there were 303 total unique supporting or opposing opinions expressed about the HPL (Fig. 5). Industry sources did not express any support for the HPL and were by far the most likely to oppose it. Government representatives expressed significantly more supporting (94%) versus opposing (6%) views (*p* < 0.001); Academics expressed significantly more supporting (97%) versus opposing (3%) views (p < 0.001). NGOs expressed significantly more supporting (82%) versus opposing (18%) views (*p* < 0.01). Private citizens expressed substantially more supporting (75%) versus opposing (25%) views, as did economists (63% supporting, 37% opposing), but these results did not reach statistical significance.

### Proposed solutions: actor responsible and level of action

Considering all sources, industry (15%) or government (73%) were the actors most commonly proposed to fix the problem of overconsumption of sugar. Industry was the most likely to propose industry voluntary actions as a solution (38.3% of mentions of this solution came from industry), followed by government sources (31.7% of mentions). Government was the most likely to propose governmental regulations as a solution (46.3%), followed by academic sources (23.8% of mentions). Only one statement was recorded, by a government official, that NGOs were a key actor responsible for solutions. Finally, governmental sources were the most likely to propose individual level decisions and behavior changes as a solution (27.7%), followed equally by industry and academic sources (23.4% of mentions) (Table 1).

**Table 1:** Who is responsible for proposed solutions to excessive sugar intake stratified by source proposing the solution

	Actor responsible for solution:	Industry voluntary (***n*** = 29)	Government regulations (***n*** = 141)	NGO(n = 1)	Individual(***n*** = 26)
**Source proposing the solution:**	Industry	38.3%^**a**^	11.2%^**b**^	0.0%	23.4%
	Government	31.7%	46.3%^**a**^	100.0%	27.7%
	Academic	16.7%^**b**^	23.8%	0.0%	23.4%
	Economist	1.7%	1.9%	0.0%	0.0%
	NGO	8.3%	12.6%	0.0%	10.6%
	Private Citizen	3.3%	4.2%	0.0%	14.9%
	Total	100.0%	100.0%	100.0%	100.0%

^a^Significantly greater percentage than expected based on overall prevalence of source

^b^Significantly lower percentage than expected based on overall prevalence of source

There was a total of 222 different proposed solutions in all articles, with 27 solutions occurring at the level of individual action, 32 occurring at the level of individual beliefs, 25 occurring at the level of food nutrition (i.e., reformulation), and 138 occurring at the level of the food environment (Table 2). There were no statistically significant differences among the five different types of sources that proposed solutions at the level of individual action. Industry was the most likely to propose solutions that operated at the level of individual beliefs and voluntary changes in food supply. The government, as well as academic and health experts, were the most likely to suggest structural changes in the food environment including taxes that could increase the price of SSBs. Industry actors were least likely to suggest structural changes in food environment to improve health (Table 2).

**Table 2:** Levels of action for proposed solutions stratified by source proposing the solution

	Level of change for solution:	Individual actions (***n*** = 27)	Individual beliefs (***n*** = 32)	Food supply (***n*** = 25)	Other food environment (***n*** = 138)
		% of Solution by Source	% of Solution by Source	% of Solution by Source	% of Solution by Source
**Source proposing the solution:**	**Industry**	27%	38%^**a**^	73%^**a**^	1%^**b**^
	**Government**	15%^**b**^	14%^**b**^	12%^**b**^	54%^**a**^
	**Academic**	24%	22%	12%^**b**^	26%
	**Economist**	0%	0%	4%	2%
	**NGO**	9%	14%	0%	14%
	**Private Citizen**	24%	14%	0%	3%
	**Total**	100%	100%	100%	100%

^a^Significantly greater percentage than expected based on overall prevalence of source

^b^Significantly lower percentage than expected based on overall prevalence of source

## Discussion

This analysis of South African newspaper articles about the April 2018 implementation of the Health Promotion Levy, a sugary drinks tax, found overall HPL coverage was highest from the month of the initial announcement to the passage of the bill in December 2017, after which coverage declined. Although we found a surge in overall coverage following the announcement of the HPL, we did not find increases in the proportion of negative views of the tax during this period. These findings differ from a study in the United Kingdom, which found a surge in articles opposing the tax in the month it was announced [17]. Following the announcement of the HPL plan in February 2016 and the implementation of the law in December 2017, we saw increases in both positive and negative articles. This finding could indicate a more receptive environment for the HPL compared to the UK beverage tax, more successful efforts by HPL proponents to be represented in the media, or an overall heightened awareness of the growing body of evidence for the health benefits of sugary beverage taxes. Indeed, an evaluation of the mass media campaign “Are You Drinking Yourself Sick?” found increases in knowledge of the link between sugary beverages and obesity as well as other poor health outcomes [40]. In terms of overall supporting or opposing articles written about the HPL, there were no significant differences in the proportion of supporting, opposing, or balanced articles before compared to after the tax was passed.

In terms of stakeholder representation, industry, government, and academics (including medical experts) were nearly equally represented as sources in newspaper articles (25, 31, and 25% of sources, respectively). When analyzing total number of supporting and opposing opinions by source, we found no evidence of industry offering supporting opinions of the HPL in any articles in our sample. In fact, almost all opposing claims about the HPL were made by representatives of companies that sell SSBs or companies in the sugar industry. On the other hand, government officials, academics and medical experts, and NGO representatives offered significantly more supporting opinions of the HPL than opposing ones. These results corroborate previous findings that among stakeholders including industry, government, public health experts, and the public, opposition from industry is a common barrier to SSB tax implementation [41].

Overall, health topics were discussed more often than economics topics (82% of articles vs. 68% of articles). Among health topics, government and academic experts were more likely than industry to draw links between SSBs or sugar and health problems (obesity, diabetes). Industry actors were more likely than government or academics to question or challenge claims that sugar consumption is related to obesity. Industry was also more likely than other sources to use questioning or counter-claims to challenge claims that the HPL would lead to improved health outcomes. This latter tactic casts doubt on public health policies aimed at improving diets and has been used in other business sectors to weaken the direct link between policy and the problem that policy is meant to address. Other studies have found unhealthy commodity industries resist regulation by arguing that singling out particular commodities is unlikely to solve the stated problem and therefore is not worth the costs [42]. In the case of opposing the HPL, if sugar is not the *only* cause of obesity, then it is unjustified to single out SSBs for regulation.

For economics topics, claims that the HPL would cause economic harm were made almost entirely by industry representatives who have a financial interest in SSB sales. These harms included harming the poor through increased SSB prices, harming vulnerable workers directly through job loss, overall harm to the South African economy, or harming the sugar and SSB production industries. Disproportionate financial burden on the poor, known as “regressivity,” is a common criticism of health-related taxes, including sugary drinks taxes but also taxes on cigarettes, alcohol, and other health-harming products [43–45]. Yet, poorer populations also tend to potentially gain the most from these taxes in terms of the potential health benefits of lower SSB consumption [43–45]. In terms of overall harm, Barnhill and King argue that, compared to the cost of soda, it is the disproportionate disease burden of low-income populations from unhealthy diets that is the more morally urgent concern [43]. Many of these claims of economic harm can be assessed in light of available evidence from other national SSB taxes. A study on the effects of SSB taxes in Mexico by income found that low income households had the greatest reductions in SSB purchases [46], suggesting that low income households would potentially receive the greatest health benefits. In South Africa, a country with high wealth inequality and unemployment, low-income populations are far less likely to be diagnosed and treated for sugar-related NCDs, making primary prevention an even higher priority. With respect to job loss, evidence from Mexico published in December 2017 suggests there was no drop in employment in either the manufacturing or food and beverage sales sectors following an SSB and nonessential food tax [47]. With respect to the overall economy, there was also no evidence of an effect on national unemployment figures. However, this evidence was published in the middle of our study sample period, so it is unclear whether the sources referenced in our study sample were aware of it to respond to this claim. Another study from the UK found a short-term negative impact on the beverage industry after the national SSB tax was announced, but these effects did not persist post-implementation. Future studies are needed to determine if the economic effects of the HPL were similar to those of the national SSB taxes in Mexico or the UK [48].

Although most economics topics were used in opposition to the tax, non-industry actors pointed to the possible economic benefits of the HPL. Consistent with extensive modeling studies from multiple countries that SSB taxes could reduce burden on health systems [49–55], governments and academics were disproportionately more likely to highlight this potential economic benefit of the HPL.

This tension between health frames and economic frames for discussing public health policies has been a consistent finding in media studies of health policy. Weishaar and colleagues [11] trace this enduring conflict back to Beauchamp [56], who identified “market justice” as an opposing force to “social justice”. In this conflict, public health seeks to protect people, particularly the vulnerable, and in doing so may infringe on corporations’ rights to sell a product or to evade responsibility for disease prevention [56]. Previous work has found these SSB policy frames may be key to influencing public opinion [41, 57–60]. In New York City, where a ban on large SSB portion sizes failed, 84% of newspaper articles studied contained opposing frames centered around economic concerns and freedom of the market compared to only 36% pro-policy frames that covered potential health benefits of the policy [61]. In other contexts, food taxes framed positively as beneficial to health corresponded with increased public support [18, 62–64]. We found similar opposing frames in our news media study of the HPL in South Africa, wherein health related frames were more likely to be utilized by government and academics in ways that were supportive of the HPL, whereas economic frames more likely to be utilized by industry in ways that were in opposition to the HPL.

Our results can inform policymakers by demonstrating that health frames are more commonly used to support SSB taxes, and economic frames are more commonly used to undermine them. However, it is notable there has been limited to no evidence of actual economic harm caused by these policies [47], whereas there is published evidence of potential health benefits from modeling studies [49–55]. With a clear understanding of frames associated with policy support, public health officials may be better prepared to generate public support.

Among the solutions for health problems related to excessive sugary beverage consumption that were proposed in the newspaper articles we analyzed, the most common solution was government regulation of the food environment. These results corroborate findings from online newspapers in the UK, where government intervention was the most commonly proposed solution for addressing sugar overconsumption [17]. Government representatives were the mostly likely to propose this intervention, followed by academics and medical experts, whereas industry representatives were the most likely to suggest their own voluntary solutions. Although it is common for food and beverage companies to commit to self-regulation, voluntary self-regulation has proven largely ineffective [65, 66]. For example, a systematic review of initiatives to limit the advertising of food and beverage products to children found that peer-reviewed academic literature was more likely to find high levels of advertising unhealthy foods despite industry commitments to self-regulation, contradicting industry-sponsored reports [67].

Our study has several limitations. First, we are unable to make causal claims about the effects of the news media on how people respond to SSB taxes. Future studies would benefit from linking news media exposure to individual-level consumption of SSBs to better understand their relationship. Another limitation is our focus on only English language sources. South Africa has great language diversity, with eleven official languages, many of which are acquired as a first language. It is therefore possible that our results are biased if coverage of the SSB tax differs between non-English and English sources. Average readership of our sources is presented in Additional File 1: Table S1. However, we cannot directly link the average monthly readership of online and offline copies of publications estimated by SAARF to specific viewership of the HPL articles we examined. A more direct measure than publication readership would be needed to evaluate actual exposure to relevant articles, such as unique viewer counts or individualized recordings of readership. Finally, although our analysis did not include other forms of media and political commentary including social media or television, a focus on newspapers has been used as a method to analyze the media response to SSB and tobacco regulations [11, 28, 29, 57, 61, 68–70]. Previous studies of the South African media landscape suggest print media readership may be skewed toward wealthy elites [71]. Other forms of online media including social media present an opportunity to capture media exposures of younger groups. Future research could benefit by examining the role of social media in shaping attitudes toward the HPL and other health promotion policies. Smartphone ownership in sub-Saharan Africa is growing but remains low, with South Africa being the only country with more than 50% of respondents report having a smartphone [72]. Among those South Africans with smartphones, social media use is steadily growing. According to Pew Research, the percentage of adults who use social networks in South Africa increased from 27% in 2013 to 43% in 2017 [73]. A recent study of adolescents and young adults living in a rural community in South Africa found approximately 70% of respondents believed Facebook was a place to share knowledge [74]. Future work could examine the types of knowledge shared and the extent to which health policy debates are had on social media platforms.

Despite these limitations, this study has several strengths. Our analysis captured the major national English language newspapers; according to the South African Audience Research Foundation (SAARF), our analysis includes 23 of the top English language newspapers in South Africa [31]. In addition, by including sources in our analyses, we are able to capture not only what topics are invoked most often but also the perspectives of key stakeholders in the news media discussion about the purpose and consequences of the HPL. This is an important inclusion as it more thoroughly characterizes the news media environment in South Africa responding to the SSB tax.

## Conclusions

This study found that industry representatives were more likely to oppose the HPL than academics, government representatives, NGOs, or economists, with the most common reason being negative economic impacts. Within all non-industry sources, a majority expressed a supportive view of the tax. The most common reasons for supporting the HPL were the link between sugar consumption and obesity and likely health benefits from the HPL. Understanding the news media response to the national sugary beverage tax may provide insight for future studies evaluating the effects of the tax on public perception of SSBs and their consumption. More studies are needed to not only characterize the media environments related to public health policies, but also to link media exposure to individual level changes in policy perception. Our study is also limited to a focus on online news articles published in English. Future work could expand an understanding of the media environment for the HPL and health promotion policies by examining changes in other media including television, radio, and social media.

## Supplementary Information

**Additional file 1: Table S1**. Frequency of articles published about the Health Promotion Levy by major South African newspapers (*n* = 193 total), from February 2016 to June 2019.

**Additional file 2: Table S2**. Codebook definitions including major categories of health, hconomics, and proposed solutions followed by definitions of sources.

## Data Availability

The datasets used and/or analysed during the current study are available from the corresponding author on reasonable request.

## Abbreviations

HPL: Health Promotion Levy
LMIC: Low- and middle- income country
NGO: Non-governmental organization
SAARF: South African Audience Research Foundation
SSB: sugar-sweetened beverages
UK: United Kingdom
