# Supplementary material for: Examining the news media reaction to a national sugary beverage tax in South Africa: a quantitative content analysis
Source: BMC Public Health. 2021 Mar 6;21:454. doi: 10.1186/s12889-021-10460-1 (PMC7937301; doi:10.1186/s12889-021-10460-1)
Supplement: Supplementary file 1 — Additional file 1: Table S1. Frequency of articles published about the Health Promotion Levy by major South African newspapers (n = 193 total), from February 2016 to June 2019. [file 12889_2021_10460_MOESM1_ESM.docx]

**Additional File 1: Table S1.** Frequency of articles published about the Health Promotion Levy by major South African newspapers (n=193 total), from February 2016 to June 2019

| **Publication name** | **Frequency** | **% of sample** | **Monthly readers*** |
| --- | --- | --- | --- |
| **Daily Newspapers** |  |  |  |
| Sowetan | 6 | 3.1 | 1482000 |
| The Star | 17 | 8.8 | 621000 |
| Daily News | 11 | 5.7 | 257000 |
| The Daily News | 2 | 1 | 257000 |
| The Times | 10 | 5.2 | 255000 |
| Daily Dispatch | 8 | 4.2 | 251000 |
| Post | 2 | 1 | 247000 |
| Cape Argus | 15 | 7.8 | 216000 |
| The Herald | 8 | 4.2 | 205000 |
| The Mercury | 20 | 10.4 | 200000 |
| Cape Times | 23 | 11.9 | 183000 |
| Pretoria News | 14 | 7.3 | 144000 |
| The New Age | 8 | 4.2 | 136000 |
| DFA | 2 | 1 | 108000 |
| Business Day | 12 | 6.2 | 79000 |
| **Weekly Newspapers** |  |  |  |
| Sunday Times | 6 | 3.1 | 3704000 |
| Sunday World | 1 | 0.5 | 1311000 |
| Mail & Guardian | 10 | 5.2 | 564000 |
| Sunday Tribune | 9 | 4.7 | 290000 |
| Argus Weekend | 1 | 0.5 | 227000 |
| The Independent on Saturday | 4 | 2.1 | 95000 |
| The Sunday Independent | 4 | 2.1 | 63000 |

*****Average Issue Readership numbers reported by South African Audience Research Foundation’s (SAARF) All Media Products Survey (Amps) Newspaper Readership and Trends for 2015 [31]. SAARF counts an average issue reader as someone who has “read or paged through any copy of the title under consideration within a period before the interview which is no longer than the issue period of that title” [75]. This table presents SAARF estimates of the number of readers who accessed any online or offline copy of a publication in the last month.
