# Supplementary material for: Examining the news media reaction to a national sugary beverage tax in South Africa: a quantitative content analysis
Source: BMC Public Health. 2021 Mar 6;21:454. doi: 10.1186/s12889-021-10460-1 (PMC7937301; doi:10.1186/s12889-021-10460-1)
Supplement: Supplementary file 2 — Additional file 2: Table S2. Codebook definitions including major categories of health, hconomics, and proposed solutions followed by definitions of sources. [file 12889_2021_10460_MOESM2_ESM.docx]

**Additional File 2: Table S2.** Codebook definitions including major categories of Health, Economics, and proposed solutions followed by definitions of Sources

| **Topic** | **Definition** |
| --- | --- |
| The HPL will reduce SSB consumption | Mark yes if there is any mention that the HPL will reduce the consumption of sugar-sweetened beverage by citizens. This is about consumers, NOT about producers. |
| The HPL will not reduce SSB consumption | Mark yes if there is any mention that the HPL will do little or nothing to reduce the consumption of sugar-sweetened beverage by South Africans. |
| The HPL will cause or has caused reformulation | Mark yes if there is any mention, specifically related to the HPL, that will cause or has caused changes in the sugar content or other ingredient content in beverages. Also includes any mention of using of non-caloric sweeteners (including alternate names, e.g., Stevia, Splenda, aspartame, Nutrisweet, artificial sweeteners, etc.). This is about consequences that have either occurred or are thought will occur in the future as a result of the HPL. |
| Support HPL | Mark yes if there is an explicit mention of support or implied support by any previous codes that suggest the country of South Africa or its citizens will benefit from the tax. It is not enough to say that SSB consumption needs to be reduced, the support needs to be explicitly for the tax. |
| Oppose HPL | Mark yes if there is an explicit mention or implication that suggests the country of South Africa or its citizens will not benefit from the tax. This can either be a statement of the government *should* not tax or that the tax is not justified, or it can be a statement saying the tax will not have the desired consequences (e.g., will not reduce obesity). |
| ***Health - any mention*** | Mark yes if there is ANY mention of health issues such as population obesity, diabetes, tooth decay, or other non-communicable diseases. Other topics that are clearly health outcomes are also acceptable. |
| Obesity is related to SSB consumption | Mark yes if there is any mention/endorsement/suggestion that SSB consumption or sugar consumption is related to obesity or weight gain. |
| Obesity is not related to SSB consumption | Mark yes if there is any mention/endorsement/suggestion that SSB consumption or sugar consumption is NOT related to obesity. For example, the article may say that sugar is merely a source of calories, and therefore SSBs have no increased risk of obesity beyond any other food. This is not the same as an absence of the topic altogether. Do not code yes merely for absence of any mention. |
| Diabetes is related to SSB consumption | Mark yes if there is any mention/endorsement/suggestion that SSB consumption or sugar consumption is related to diabetes. |
| NCDs related | Mark yes if there is any mention/endorsement/suggestion that SSB consumption or sugar consumption is related to NCDs (other than obesity, diabetes, dental problems). |
| The HPL will improve health outcomes | Mark yes if there is any mention that the HPL has had, or will have, a beneficial effect on health outcomes (obesity, diabetes, dental, or other NCDs). This code is about health-related outcomes, or measurable health improvements. We are not including behaviors such as reduced SSB consumption. |
| The HPL will not improve health outcomes | Mark yes if there is any mention that the HPL has not had, will not have, or has unlikely or uncertain benefits to health outcomes (obesity, diabetes, dental, or other NCDs). Mark yes if the author or source says that the HPL will not improve health in the short or in the long term. |
| ***Economic/Pricing - any mention*** | Mark yes if there is any mention of economy or economic issues, including sales of beverages, how the revenue that beverage companies make will be affected (for example, if sales decrease). Mark yes also if there is any mention of other related industries (for example, sugar cane farming) will be affected by the HPL. Also mark yes if INDIVIDUALS will be financially affected by the HPL. |
| The HPL will economically harm the poor | Mark yes if there is any mention that the HPL will in any way harm the economic situation of the poor (for example, they will suffer a greater economic burden from the HPL compared to other groups because they will still purchase SSBs but spend even more money). |
| The HPL will cause industry or economic harm | Mark yes if there is any mention that the sugar sweetened beverage industry or beverage selling companies have been or will be harmed by the HPL. Also mark yes if there is any mention of economic harm specific to related industries (but not SSB manufacturers/sellers), such as sugar cane growers, or wider negative economic effects of the HPL (e.g., inflation). If there is no specific industry indicated (for example, just says it will lead to job losses), mark yes here. The previous code, SSB industry harm, is for that specific industry. |
| The HPL will not harm industry | Mark yes if there is a refutation of the argument that the HPL will cause harm to either the SSB industry or to other industries (including farmers and other workers in the SSB supply chain). This includes any statement that says there will be limited to no economic harm as a result of the tax. |
| The HPL will reduce health care costs | Mark yes if there is a mention of economic consequences that will help reduce the burden of health care costs in the short or long term (for example, the HPL will reduce the number of overweight/obese or diabetic in the population, which will reduce health care costs). |
| ***Proposed Solutions*** | Does the article propose solutions for any of the health challenges mentioned earlier (too much sugar consumption, too much obesity, diabetes, NCDs, dental caries)? Mark 1 if yes, if no solutions proposed, then skip entire section. |
| Individual actions | Is a personal action proposed? Include diet changes, exercise changes, seeking education or counseling or other health information. |
| Individual beliefs | Is an intervention, initiative, or structural response proposed that is intended to change how individuals think of food? Include public health campaigns, educational initiatives, marketing restrictions, etc. Labels would also qualify if they were providing information. If reformulation also happens, that is a different category. |
| Changes in food supply | Is an environmental or structural measure proposed that changes the food directly? Include nutrient thresholds/limits and reformulation. Industry agreements to reformulate would count in this category, with industry the most responsible. |
| Other food environment changes | Is an environmental or structural measure proposed that changes the availability of foods or the price of foods? Include school food restrictions, taxes, subsidies, restrictions on using financial assistance programs to purchase foods, etc. |
| **Level of Intervention** |  |
| Individual actions | Changes in individual actions include diet changes, exercise changes, seeking education or other health information. |
| Individual beliefs | Changes in individual beliefs about food include public health campaigns, educational initiatives, marketing restrictions, and nutrition labels. |
| Food supply | Changes in the food supply include nutrient thresholds/limits and industry agreements to reformulate. |
| Other food environment | Other changes in the food environment include school food restrictions, taxes, and subsidies. |
| **Sources** | **Definition** |
| Industry | The source of the information, either directly stating or being quoted works for a sugary beverage company or another business firm whose profits may be affected by the HPL |
| Government | The source of the information, either directly stating or being quoted is from a member of the South African Government or the statement is from a government body. |
| Academics and Health Experts | The source of the information, either directly stating or being quoted is from a person with an academic job at a college or university. This should also include medical doctors, nurses, or other health professionals. |
| Economist | The source of the information, either directly stating or being quoted is from a source who is quoted as an economist or employee of a contract research organization conducting economics research. |
| NGO | The source of the information, either directly stating or being quoted is from a nongovernmental organization (e.g., World Health Organization) |
| Public Citizen | The source of the information, either directly stating or being quoted is a South African citizen who does not belong in any of the other above categories |
